# Nigerian secondary school adolescents’ perspective on abstinence-only sexual education as an effective tool for promotion of sexual health

**DOI:** 10.12688/f1000research.2-86.v2

**Published:** 2013-12-04

**Authors:** Mfrekemfon P Inyang, Obonganyie P Inyang

**Affiliations:** 1 Department of Human Kinetics and Health Education, Faculty of Education, University of Port-Harcourt, Rivers State, Nigeria; 2 Medical and Health Centre, University of Debrecen, Debrecen, 4032, Hungary

## Abstract

The success of any type of sexual education programme depends on the knowledge and preparedness for practice by adolescents. A recent study has found that an ‘abstinence-only’ sexual education programme is effective in reducing sexual activity among adolescents. Knowledge of abstinence-only sexual education and preparedness for practice as an effective tool for promotion of sexual health among Nigerian secondary school adolescents was studied. An analytic descriptive survey design was used for the study. The research population comprised of all public secondary schools in three southern geopolitical zones of the Niger Delta Region of Nigeria. A multistage sampling technique was used to select 2020 senior secondary school (SS1-SS3) students as sample for the study. A partially self-designed and partially adapted questionnaire from an 'abstinence-only versus comprehensive sex education' debate, from debatepedia (http://wiki.idebate.org/), entitled 'Questionnaire on Nigerian Secondary School Adolescents’ Perspective on Abstinence-Only Sexual Education (QNSSAPAOSE)' was used in eliciting information from respondents. Hypotheses were formulated and tested. Frequency counts, percentage and Pearson Product Moment Correlation were used in analysing data. A greater proportion of secondary school adolescents in this study lacked knowledge of sexual education. About 80% of the respondents could not define sexual education. The general perspective on abstinence-only sexual education was negative, as revealed by the larger number of respondents who demonstrated unwillingness to practice abstinence-only sexual education. Specifically, of those who responded in favour of abstinence-only sexual education, the youngest group of adolescents (11-13 years) and the male respondents were more likely to accept this type of education than the other groups. Poor knowledge of sexual education could be responsible for unwillingness to practice abstinence-only sexual education. Sexual education should, therefore, be introduced into the secondary school curriculum and taught by well-prepared teachers to enable an informed decision on practice.

## Introduction

Sexual education is a lifelong process of acquiring information on sex and forming attitudes, values and beliefs. It involves sexual development, sexual and reproductive health, interpersonal relationships, affection and intimacy ^1^. Abstinence-only sexual education teaches the adolescents to abstain from premarital sexual intercourse because of the advantages it offers. Such advantages include prevention of unintended pregnancies and prevention of contracting HIV/AIDS and other sexually transmitted infections. According to the Federal Law of the United States of America (USA), every school-aged child should not engage in sexual activity ^2, 3^. The USA Federal law further advocates sexual activity within the confines of monogamous marital relationship to guard against adverse psychological and physical effects associated with premarital sexual activity. Bearing children outside marriage has serious effects on the child, the mother and the society as a whole ^3– 6^. The Federal Law of United States of America also posited that, abstaining from sexual activity outside marriage allows for maturity and understanding of self ^3, 7^.

Studies disclose that it is comprehensive sexual education and not abstinence-only that will delay first sexual activity ^2, 3, 8– 14^. Abstinence-only sexual education lacks strong evidence of effectiveness because of faulty designs ^3, 15, 16a^. Abstinence-only sexual education does not positively affect the sexual behaviour of adolescents, lacks the message of sexually transmitted infections to its recipients and the positive effect in a few cases does not last for a long time ^16b, 17^.

According to Bruckner and Bearman ^18^ and a study by the Alan Guttmacher Institute ^19^ those that embrace abstinence-only sexual education still have sex before they get married. There is no difference between adolescents that accept abstinence-only sexual education and those who do not in terms of number of sexual partners and ages of first sexual intercourse ^17^. Abstinence-only sexual education does not reduce the scourge of HIV/AIDS ^16b^. Bennett and Assefi saw the failure to provide adolescents with information about contraception as a serious weakness of abstinence only sexual-education ^20^.

The proponents of abstinence-only sexual education frown at the role of comprehensive sexual education in emphasising so much on the reliability of contraceptives while de-emphasising their failure rates and the possibility of contracting new sexually transmitted diseases including HIV/AIDS ^21^. They further frowned at the double message of comprehensive sexual education such as encouraging the delay of first sexual intercourse and promoting the use of contraceptives ^22^. They are also blamed for stressing the possibility of contracting sexually transmitted infections to the extent of falsifying information to establish the negative aspect of comprehensive sexual education ^1, 23, 24^. Studies further reveal that sexual educators do not stress enough on sexual intercourse or bring in sensitive issues such as homosexuality and abortions. The adolescents posited that the basic message is that they should not have sex ^19, 25– 27^. The proponents of comprehensive sexual education attributed the ineffectiveness of condoms and contraceptives to poor-quality research ^28^.

Abstinence-only sexual health education is also blamed for withholding information on the positive aspects of sexual relationships, while magnifying the emotional risks and pitfalls associated with premarital sexual activity ^1, 24^. Modern lifestyle is characterised by a high rate of broken marriages, predisposing an individual to having many sexual partners ^1^. According to some studies the age of first marriage has risen to 30 years, with a fifth of such marriages ending in divorce within a period of five years ^29^. Conversely the age at first sexual intercourse has dropped to 16 years with very few people having their husbands as their first sexual partners ^30, 31^. The data on adolescents’ sexual behaviour in the developing world with a high prevalence of HIV/AIDS suggests unacceptability of abstinence-only sexual education. Some countries do not accept abstinence-only sexual education even in the face of the high prevalence of HIV/AIDS. Some countries expect sexual educators to encourage adolescents to delay their first sexual experience while also providing education on contraception and sexual health services ^32^. In some countries, the requirements for teaching any type of sexual education are clearly outlined for the sexual educators ^33^.

Most studies reveal comprehensive sexual education as the preference of parents and adolescents ^34– 36^. Studies advocate comprehensive sexual education for unmarried sexually active adolescents ^3^. Studies also recommend abstinence-only sexual education with information on contraception and risk-reduction behaviour for the few sexually inactive adolescents. These align with the position of Collins and Priya that parents and adolescents prefer comprehensive sexual education to abstinence-only sexual education ^37^. Most studies revealed that the opinion of adults differs on the type of sexual education to teach adolescents. Most of the adults feel 7 ^th^ to 9 ^th^ graders should be taught ‘abstinence only’ while some advocate the teaching of contraception use ^38^. Most studies also show that a greater percentage of adolescents prefer sexual orientation that will teach them about the use of contraception and sexually transmitted infections than abstinence-only sexual education ^39^. Other studies reported the positive outcome of abstinence-only sexual education ranging from reduced sexual activity, pregnancies, abortions to more successful deliveries ^40^. In line with this, John and Jemmott disclosed the success of abstinence-only sexual education in reducing sexual activity among youths ^41^. In support of the findings of this new study, Rector submitted that out of 15 scientific evaluations of abstinence-only sexual education, 11 of them demonstrated its effectiveness in reducing adolescent sexual activity ^42^.

A survey of the National Campaign to Prevent Teen Pregnancy in 2001 showed that 93% of abstinence sexual education came from the society ^43^. They concluded that abstinence-only sexual education is the only 100% effective method to prevent teenage pregnancy and sexually transmitted diseases ^4^. They further reminded that condoms cannot provide 100% protection against unplanned pregnancy and sexually transmitted infections and also that premarital sex can lead to life threatening health problems such as abortion and its associated complications ^5^. They feel burdened that sexual, contraceptive and HIV information can provoke early sexual initiation among the adolescents ^5^. Abstinence-only sexual education has positively produced a corresponding decrease in teenage pregnancy ^4^. Studies have demonstrated that religion acts as a deterrent to early sexual activity ^5^. In line with this, many adolescents submitted that morals, values and religious beliefs significantly influence the decision to have sex or not.

In deciding whether or not to have sex, the Organization of Concerned Women for America also decried the outcome of sex without love or responsibility supported by public policies. This results in the breakdown of nuclear families, increases crime, poverty, teen births and AIDS which in turn negatively affects the health of the general public. This only shows lack of values ^5^.

## Statement of the problem

Adolescents are the future and so they require proper guidance that will propel them into responsible productive adults useful to themselves and their nations. Adolescence is a remarkable period characterised by the quest for experimentations with drugs, alcohol and sexual activity saddled with numerous life threatening adverse effects. The Nigerian Association for the Promotion of Adolescent Health and Development, (NAPAHD) found that, a hospital based research study revealed that 80% of patients with abortion complications in hospitals are adolescents. Studies in Nigeria have also shown that most female adolescents by the age of 15 have already had their first sexual intercourse ^6, 45^. The same applies to male adolescents. In Nigeria, complicated abortion, sexually transmitted infections and HIV/AIDS, sexual coercion, unplanned and unwanted sexual activity and unwanted pregnancies and babies, drop outs from schools and homelessness abound and are very common features with the adolescents ^6^. Most Nigerian adolescents do not receive correct sexual information while some are ignorant. Hold back their potentials and also affect the nation negatively ^6^. The intense outcome associated with adolescent sexual activity necessitates the search for a positive way out and thus, the main objective of this study.

## Purpose of the study

According to Focus on the Family group, sex should be avoided the same way as the use of guns, tobacco, alcohol and drink-driving ^46^. They sternly condemn the advocacy for the use of condoms against unwanted pregnancies and sexually transmitted diseases in favour of abstinence-only sexual education ^47^. Previous studies document the advantages of abstinence-only sexual education in reducing adolescent sexual activities and the associated health problems. This study investigated the perspective of secondary school adolescents in Nigeria on abstinence-only sexual education as an effective tool for promoting adolescent sexual health. The findings will help in planning informed corresponding intervention programmes.

## Material and methods

An analytic descriptive survey design was used for the study. The research population comprised of all public secondary schools in three southern geopolitical zones of the Niger Delta Region of Nigeria. The States were Rivers, Akwa Ibom and Cross River. A multistage sampling technique was used for selecting 2020 senior secondary school (SS1-SS3) male and female students from the three states. Proportionate sampling technique was used in selecting the number of participating schools from the metropolis of each state. Five schools were selected from each of Cross River and Akwa Ibom States. Ten schools were selected from Rivers State. Respondents from each school were also selected proportionately. A total of 702 respondents were drawn from Akwa Ibom State, 510 from Cross River State and 808 from Rivers State. Participants were within the age range of 10–19 years. A questionnaire entitled Questionnaire on Nigerian Secondary School Adolescents Perspective on Abstinence-Only Sexual Education (QNSSAPAOSE) was used in eliciting information from respondents. The test/re-test reliability method was used to establish the internal consistency of the instrument. The instrument had a reliability coefficient of 0.75 established with Pearson Product Moment Correlation Coefficient (r). The instrument was divided into sections A and B. Section ‘A’ sought information on respondents’ demographic characteristics. Section ‘B’ sought information on respondents’ perspectives on Abstinence-Only sexual education. Questions were closed and open ended. The YES/NO questions attracted two points for positive responses and one point for negative responses. Questionnaires were administered with the aid of ten trained research assistants. Questionnaires were administered and collected on the spot to enhance a high return rate. Completion of the questionnaire was voluntary. Out of a total of 2020 questionnaires sent out, 2013 were returned and 13 questionnaires were not usable yielding a usable number of 2000 questionnaires. The return rate, therefore, was 99.1% (2000/2020). Hypotheses were formulated and tested. Frequency counts, percentage, Standard Deviation and Pearson Product Moment Correlation were used in analysing data. Approval of individual school management was obtained prior to execution of this study in their schools. Participants’ consent was also obtained. Participation was voluntary and anonymity was also assured and maintained.

## Results

All of the participants in this study were adolescents between the ages of 10 and 19 years ( Table 1).

**Table 1.** Demographic characteristics of study participants by frequency and percentiles.

Variable	N	(%)		N	(%)
**Age**			**Class**		
11–13 years	176	8.8	SS1	1413	70.7
14–16 years	1223	61.15	SS2	486	24.3
17–19 years	601	30.05	SS3	101	5.1
Total	2000	100.0	Total	2000	100.0
Gender	N	(%)	Religion	N	(%)
Male	702	35.1	Christianity	1877	93.9
Female	1298	64.9	Islam	86	4.3
Total	2000	100.0	Pagan	31	1.6
**Ethnicity**			Traditional	6	0.3
Ikwerre	800	40	Total	2000	100.0
Ibibios	700	35			
Efiks	500	25			
Total	2000	100.0			

Three research questions and six hypotheses were formulated in this study. Each one of them is addressed as follows:

### What are the perspectives of adolescents on the advantages of abstinence-only sexual education?

Seven positive statements representing the advantages of abstinence-only sexual education were made ( Table 2). The general perspective of Nigerian secondary adolescents on the advantages of abstinence-only sexual education was negative. A greater number of the respondents did not agree with the statements highlighting the advantages of abstinence-only sexual education. However, taking into consideration the number of respondents from each age group that agreed with the statements highlighting the advantages of abstinence-only sexual education, it was found that the youngest age group (11–13 years) of adolescents ranked first. This implies that this group had the highest number of positive respondents in line with their total number when compared with the young (17–19 years) and younger (14–16 years) groups. Age group 11–13 years therefore demonstrated the likelihood of accepting abstinence-only sexual education.

**Table 2.** Perspective of adolescents according to age group on the advantages of abstinence-only sexual education.

**Item 1**	Abstinence-only sexual education promotes responsible sexual culture					
Age groups	YES		NO		TOTAL	
	N	(%)	N	(%)	N	(%)
11–13 years	62	35.2	114	64.8	176	100
14–16 years	212	17.3	1011	82.7	1223	100
17–19 years	79	13.1	522	86.9	601	100
Total	353	17.7	1647	82.4	2000	100
**Item 2**	Abstinence-only sexual education promotes healthy fulfilling relationship					
Age groups	YES		NO		TOTAL	
	N	(%)	N	(%)	N	(%)
11–13 years	89	50.6	87	49.4	176	100
14–16 years	168	13.7	1055	86.3	1223	100
17–19 years	170	28.3	431	71.7	601	100
Total	427	21.4	1573	78.7	2000	100
**Item 3**	Abstinence-only sexual education discourages youth sex and risk taking					
Age groups	YES		NO		TOTAL	
	N	(%)	N	(%)	N	(%)
11–13 years	78	44.3	98	55.7	176	100
14–16 years	280	22.9	943	77.1	1223	100
17–19 years	157	26.1	444	73.9	601	100
Total	515	25.8	1485	74.3	2000	100
**Item 4**	Abstinence-only sexual education helps youth avoid emotional damage of sex					
Age groups	YES		NO		TOTAL	
	N	(%)	N	(%)	N	(%)
11–13 years	72	40.9	104	59.1	176	100
14–16 years	186	15.2	1037	84.8	1223	100
17–19 years	120	20.0	481	80.0	601	100
Total	378	18.9	1622	81.1	2000	100
**Item 5**	Abstinence-only sexual education helps discourage out-of wedlock pregnancies					
Age groups	YES		NO		TOTAL	
	N	(%)	N	(%)	N	(%)
11–13 years	65	36.9	111	63.1	176	100
14–16 years	313	25.6	910	74.4	1223	100
17–19 years	174	29.0	427	71.0	601	100
Total	552	27.6	1448	72.4	2000	100
**Item 6**	Abstinence-only sexual education is very good in preventing sexually transmitted infections (STIs) because condoms are not effective at protecting against STIs					
Age groups	YES		NO		TOTAL	
	N	(%)	N	(%)	N	(%)
11–13 years	58	33.0	118	67.0	176	100
14–16 years	171	14.0	1052	86.0	1223	100
17–19 years	84	14.0	517	86.0	601	100
Total	313	15.7	1687	84.4	2000	100
**Item 7**	Abstinence-only sexual education effectively reduces rate of teen sex and pregnancy					
Age groups	YES		NO		TOTAL	
	N	(%)	N	(%)	N	(%)
11–13 years	59	33.5	117	66.5	176	100
14–16 years	215	17.6	1008	82.4	1223	100
17–19 years	170	28.3	431	71.7	601	100
Total	444	22.2	1556	77.8	2000	100

### What are the perspectives of adolescents on the disadvantages of abstinence-only sexual education?

The same number of statements was also made on the disadvantages of abstinence-only sexual education ( Table 3). Generally, more respondents agreed with five while disagreeing with the last two of the seven statements highlighting disadvantages of abstinence-only sexual education. Of the total number of respondents that disagreed with the statements of disadvantages, the young adolescents (17–19 years) ranked first followed by the younger ones (14–16 years). More of the young adolescents out of their total number disagreed with the statements of disadvantages when compared with the responses of the other two groups. This might be a factor of a better understanding than the younger age groups. Moreover, the problem might not be with the type of sexual education but mostly the willingness to practice.

**Table 3.** Perspective of adolescents according to age group on the disadvantages of abstinence-only sexual education.

**Item 8**	Abstinence-only sexual education sometimes encourages oral and anal sex as alternatives					
Age groups	YES		NO		TOTAL	
	N	(%)	N	(%)	N	(%)
11–13 years	98	55.7	78	44.3	176	100
14–16 years	562	46.0	661	54.0	1223	100
17–19 years	248	41.3	353	58.7	601	100
Total	908	45.4	1092	54.6	2000	100
**Item 9**	Abstinence-only sexual education is Not effective at reducing teen sex rate					
Age groups	YES		NO		TOTAL	
	N	(%)	N	(%)	N	(%)
11–13 years	111	63.1	65	36.9	176	100
14–16 years	557	45.5	666	54.5	1223	100
17–19 years	278	46.3	323	53.7	601	100
Total	946	47.3	1054	52.7	2000	100
**Item 10**	Abstinence-only sexual education discourages condom use and increases risk					
Age groups	YES		NO		TOTAL	
	N	(%)	N	(%)	N	(%)
11–13 years	89	50.6	87	49.4	176	100
14–16 years	584	47.8	639	52.2	1223	100
17–19 years	284	47.3	317	52.7	601	100
Total	957	47.9	1043	52.2	2000	100
**Item 11**	Abstinence-only sexual education does Not help decrease HIV infection rate					
Age groups	YES		NO		TOTAL	
	N	(%)	N	(%)	N	(%)
11–13 years	77	43.8	99	56.3	176	100
14–16 years	587	48.0	636	52.0	1223	100
17–19 years	261	43.4	340	56.6	601	100
Total	925	46.3	1075	53.8	2000	100
**Item 12**	Condoms and Not abstinence-only sexual education decrease teenage pregnancy					
Age groups	YES		NO		TOTAL	
	N	(%)	N	(%)	N	(%)
11–13 years	113	64.2	63	35.8	176	100
14–16 years	363	29.7	860	70.3	1223	100
17–19 years	249	41.4	352	58.6	601	100
Total	725	36.3	1275	63.8	2000	100
**Item 13**	Telling teens to abstain from sex makes them want it more					
Age groups	YES		NO		TOTAL	
	N	(%)	N	(%)	N	(%)
11–13 years	97	55.1	79	44.9	176	100
14–16 years	623	50.9	600	49.1	1223	100
17–19 years	286	47.6	315	52.4	601	100
Total	1006	50.3	994	49.7	2000	100
**Item 14**	Abstinence-only sexual education wrongly teaches suppression of sexual impulses					
Age groups	YES		NO		TOTAL	
	N	(%)	N	(%)	N	(%)
11–13 years	128	72.7	48	27.3	176	100
14–16 years	673	55.0	550	45.0	1223	100
17–19 years	293	48.8	308	51.2	601	100
Total	1094	54.7	906	45.3	2000	100

### What are the perspectives of adolescents on the acceptance of abstinence-only sexual education?

The general perspective of the respondents to five statements representing the acceptance of abstinence-only sexual education was negative. More respondents were against abstinence-only sexual education demonstrating a negative perspective on the acceptance of abstinence-only sexual education ( Table 4). For instance 1686 out of the total respondents of 2000 objected to wanting a strong abstinence-only message. The youngest adolescent group (11–13 years) had the highest number of respondents out of their total number of those in favour of abstinence-only sexual education. For instance, more of them when compared with other age groups wanted sex to be saved until marriage. More of them (11–13 years) also wanted a strong abstinence message and education. This indicates that the youngest group of adolescents were more likely to accept abstinence-only sexual education than other age groups.

**Table 4.** Perspective of adolescents according to age group on the acceptance of abstinence-only sexual education.

**Item 15**	Abstinence-only sexual education provides some information on STIs and contraception					
Age groups	YES		NO		TOTAL	
	N	(%)	N	(%)	N	(%)
11–13 years	71	40.3	105	59.7	176	100
14–16 years	307	25.1	916	74.9	1223	100
17–19 years	141	23.5	460	76.5	601	100
Total	519	26.0	1481	74.1	2000	100
**Item 16**	Abstinence-only does Not have value					
Age groups	YES		NO		TOTAL	
	N	(%)	N	(%)	N	(%)
11–13 years	137	77.8	39	22.2	176	100
14–16 years	843	68.9	380	31.1	1223	100
17–19 years	373	62.1	228	37.9	601	100
Total	1006	50.3	994	49.7	2000	100
**Item 17**	Abstinence-only sexual education is for religious people and Not for people like us					
Age groups	YES		NO		TOTAL	
	N	(%)	N	(%)	N	(%)
11–13 years	125	71.0	51	29.0	176	100
14–16 years	895	73.2	328	26.8	1223	100
17–19 years	421	70.0	180	30.0	601	100
Total	1441	72.1	559	28.0	2000	100
**Item 18**	I like abstinence-only because it advocates that sex should be saved until marriage					
Age groups	YES		NO		TOTAL	
	N	(%)	N	(%)	N	(%)
11–13 years	77	43.8	99	56.3	176	100
14–16 years	135	11.0	1088	89.0	1223	100
17–19 years	148	24.6	453	75.4	601	100
Total	360	18.0	1640	82.0	2000	100
**Item 19**	I want a strong abstinence message and education					
Age groups	YES		NO		TOTAL	
	N	(%)	N	(%)	N	(%)
11–13 years	59	33.5	117	66.5	176	100
14–16 years	168	13.7	1055	86.3	1223	100
17–19 years	87	14.5	514	85.5	601	100
Total	314	15.7	1686	84.3	2000	100

**Table 5.** Descriptive analysis of age and gender distribution.

Age	Gender				Total	
	Male		Female			
	N	(%)	N	(%)	N	(%)
11–13 years	105	59.7	71	40.3	176	100.0
14–16 years	389	31.8	834	68.2	1223	100.0
17–19 years	208	34.6	393	65.4	601	100.0
Total	702	35.1	1298	64.9	2000	100.0

### Is there any significant relationship between age and perspective on abstinence-only sexual education?

A significant relationship existed between age and the students’ perspective on abstinence-only sexual education. (r = 0.123**, N = 2000, P < 0.01) ( Table 7). Young age specifically had an influence on the respondents’ perspective on abstinence-only sexual education in this study. Null hypothesis is rejected.

### Is there any significant relationship between religion and perspective on abstinence-only sexual education?

Respondents belonged to different religious organisations but only 31 out of the total respondents of 2000 were pagans ( Table 6). A greater proportion were Christians which numbered up to 1877 out of 2000 total number of respondents. Muslims were 86 while traditional worshippers were only 6. According to the total number of each age group, the highest number of Christians came from age group 14–16 years followed by age group 17–19. Age group 11–13 years which has demonstrated the likelihood of accepting abstinence-only sexual education had the least number of Christians relatively.

**Table 6.** Descriptive analysis of age and religion distribution.

Age	Religion								Total	
	Christian		Muslim		Pagan		Traditional			
	N	(%)	N	(%)	N	(%)	N	(%)	N	(%)
11–13 years	159	90.3	7	4.0	10	5.7	-	-	176	100.0
14–16 years	1165	95.3	34	2.8	21	1.7	3	0.2	1223	100.0
17–19 years	553	92.0	45	7.5	-	-	3	0.5	601	100.0
Total	1877	93.9	86	4.3	31	1.6	6	0.3	2000	100.0

**Null hypothesis 1:** There is no significant relationship between age and their perspective on abstinence-only sexual education.

**Table 7.** Relationship between age and perspective on abstinence-only sexual education.

Variable	Mean	Std. Dev.	N*	R***	P****	Remark
Perception of abstinence-only sexual education on	31.2350	3.5702	2000	.123**	.000	Sig.
Age	15.6390	1.7565				

*No of participants.

**Sig. at 0.01 level.

***Pearson’s.

****'p' value.

**Table 8.** Perspective of adolescents according to gender on the advantages of abstinence-only sexual education.

**Item 1**	Abstinence-only sexual education promotes responsible sexual culture					
Gender	YES		NO		TOTAL	
	N	(%)	N	(%)	N	(%)
Male	120	17.1	582	82.9	702	100
Female	233	18.0	1065	82.0	1298	100
Total	353	17.7	1647	82.4	2000	100
**Item 2**	Abstinence-only sexual education promotes healthy fulfilling relationship					
Gender	YES		NO		TOTAL	
	N	(%)	N	(%)	N	(%)
Male	191	27.2	511	72.8	702	100
Female	236	18.2	1062	81.8	1298	100
Total	427	21.4	1573	78.7	2000	100
**Item 3**	Abstinence-only sexual education discourages youth sex and risk taking					
Gender	YES		NO		TOTAL	
	N	(%)	N	(%)	N	(%)
Male	192	27.4	510	72.6	702	100
Female	323	24.9	975	75.1	1298	100
Total	515	25.8	1485	74.3	2000	100
**Item 4**	Abstinence-only sexual education helps youth avoid emotional damage of sex					
Gender	YES		NO		TOTAL	
	N	(%)	N	(%)	N	(%)
Male	166	23.6	536	76.4	702	100
Female	212	16.3	1086	83.7	1298	100
Total	378	18.9	1622	81.1	2000	100
**Item 5**	Abstinence-only sexual education helps discourage out-of wedlock pregnancies					
Gender	YES		NO		TOTAL	
	N	(%)	N	(%)	N	(%)
Male	182	25.9	520	74.1	702	100
Female	370	28.5	928	71.5	1298	100
Total	552	27.6	1448	72.4	2000	100
**Item 6**	Abstinence-only sexual education is very good in preventing sexually transmitted infections (STIs) because condoms are Not effective at protecting against STIs					
Gender	YES		NO		TOTAL	
	N	(%)	N	(%)	N	(%)
Male	127	18.1	575	81.9	702	100
Female	186	14.3	1112	85.7	1298	100
Total	313	15.7	1687	84.4	2000	100
**Item 7**	Abstinence-only sexual education effectively reduces rate of teen sex and pregnancy					
Gender	YES		NO		TOTAL	
	N	(%)	N	(%)	N	(%)
Male	135	19.2	567	80.8	702	100
Female	309	23.8	989	76.2	1298	100
Total	444	22.2	1556	77.8	2000	100

**Table 9.** Perspective of adolescents according to gender on the disadvantages of abstinence-only sexual education.

**Item 8**	Abstinence-only sexual education sometimes encourages oral and anal sex alternatives					
Gender	YES		NO		TOTAL	
	N	(%)	N	(%)	N	(%)
Male	317	45.2	385	54.8	702	100
Female	591	45.5	707	54.5	1298	100
Total	908	45.4	1092	54.6	2000	100
**Item 9**	Abstinence-only sexual education is Not effective at reducing teen sex rate					
Gender	YES		NO		TOTAL	
	N	(%)	N	(%)	N	(%)
Male	338	48.1	364	51.9	702	100
Female	608	46.8	690	53.2	1298	100
Total	946	47.3	1054	52.7	2000	100
**Item 10**	Abstinence-only sexual education discourages condom use and increases risk					
Gender	YES		NO		TOTAL	
	N	(%)	N	(%)	N	(%)
Male	351	50.0	351	50.0	702	100
Female	606	46.7	692	53.3	1298	100
Total	957	47.9	1043	52.2	2000	100
**Item 11**	Abstinence-only sexual education does Not help decrease HIV infection rate					
Gender	YES		NO		TOTAL	
	N	(%)	N	(%)	N	(%)
Male	313	44.6	389	55.4	702	100
Female	612	47.1	686	52.9	1298	100
Total	925	46.3	1075	53.8	2000	100
**Item 12**	Condoms and Not abstinence-only sexual education decrease teenage pregnancy					
Gender	YES		NO		TOTAL	
	N	(%)	N	(%)	N	(%)
Male	242	34.5	460	65.5	702	100
Female	483	37.2	815	62.8	1298	100
Total	725	36.3	1275	63.8	2000	100
**Item 13**	Telling teens to abstain from sex makes them want it more					
Gender	YES		NO		TOTAL	
	N	(%)	N	(%)	N	(%)
Male	394	56.1	308	43.9	702	100
Female	612	47.1	686	52.9	1298	100
Total	1006	50.3	994	49.7	2000	100
**Item 14**	Abstinence-only sexual education wrongly teaches suppression of sexual impulses					
Gender	YES		NO		TOTAL	
	N	(%)	N	(%)	N	(%)
Male	381	54.3	321	45.7	702	100
Female	713	54.9	585	45.1	1298	100
Total	1094	54.7	906	45.3	2000	100

**Table 10.** Perspective of adolescents according to gender on the acceptance of abstinence-only sexual education.

**Item 15**	Abstinence-only sexual education provides some information on STIs and contraception					
Gender	YES		NO		TOTAL	
	N	(%)	N	(%)	N	(%)
Male	175	24.9	527	75.1	702	100
Female	344	26.5	954	73.5	1298	100
Total	519	26.0	1481	74.1	2000	100
**Item 16**	Abstinence-only does Not have value					
Gender	YES		NO		TOTAL	
	N	(%)	N	(%)	N	(%)
Male	477	67.9	225	32.1	702	100
Female	876	67.5	422	32.5	1298	100
Total	1353	67.7	647	32.4	2000	100
**Item 17**	Abstinence-only sexual education is for religious people and Not for people like us					
Gender	YES		NO		TOTAL	
	N	(%)	N	(%)	N	(%)
Male	498	70.9	204	29.1	702	100
Female	943	72.7	355	27.3	1298	100
Total	1441	72.1	559	28.0	2000	100
**Item 18**	I like abstinence-only because it advocates that sex should be saved until marriage					
Gender	YES		NO		TOTAL	
	N	(%)	N	(%)	N	(%)
Male	171	24.4	531	75.6	702	100
Female	189	14.6	1109	85.4	1298	100
Total	360	18.0	1640	82.0	2000	100
**Item 19**	I want a strong abstinence message and education					
Gender	YES		NO		TOTAL	
	N	(%)	N	(%)	N	(%)
Male	154	21.4	548	78.1	702	100
Female	160	12.3	1138	87.7	1298	100
Total	314	15.7	1686	84.3	2000	100

**Null hypothesis 2:** There is no significant relationship between gender and their perspective on abstinence-only sexuality education.

### Gender and perspective on abstinence-only sexual education?

A greater number of male and female adolescents demonstrated a negative perspective to the advantages of abstinence-only sexual education. This is deduced from their responses to the statements reflecting the advantages of abstinence-only sexual education. Out of the total number according to gender that responded in favour of the advantages of abstinence-only sexual education, more females were in favour of three statements while more males were in favour of four statements.

More male and female respondents disagreed with five of the statements reflecting the disadvantages of abstinence-only sexual education and agreed with two of the statements. Out of the total number of those that did not agree with the disadvantages of abstinence-only sexual education, more males responded to four of the statements while more females responded to three of the statements.

An outright negative perspective on the acceptance of abstinence-only sexual education was demonstrated by both male and female adolescents. A greater number of male and female respondents reacted negatively to statements which were in favour of abstinence-only sexual education.

More females responded in favour of acceptance of abstinence-only sexual education in two statements out of five. More males responded to three of the statements. Specifically, more males than females advocated for sex to be saved until marriage and also wanted a strong abstinence message and education. This study found that boys are more likely to accept abstinence-only sexual education than females. This finding might be connected with the larger number of males among age 11–13 year group of adolescents with a likelihood to accepting abstinence-only sexual education than other older adolescents ( Table 16).

There was no significant relationship between gender and perspective of the adolescents on abstinence-only sexual education. (r = 0.051, N = 2000, P < 0.05) ( Table 11). Gender had no influence on perspective of abstinence-only sexual education in the study. Null hypothesis is therefore retained.

**Table 11.** Relationship between gender and perspective on abstinence-only sexual education.

Variable	Mean	Std. Dev.	N**	R***	P****	Remark
Perception of abstinence-only sexual education on	31.2573	3.5702	2000	0.022	0.051*	N.Sig.
Gender	01.6500	0.4800				

*Not. Significant. at 0.05 level.

**No of participants.

***Pearson’s.

****'p' value.

**Null hypothesis 3:** There is no significant relationship between religion and their perspective on abstinence-only sexual education.

**Table 12.** Relationship between religion and perspective on abstinence-only sexual education.

Variable	Mean	Std. Dev.	N*	R***	P****	Remark
Perception of abstinence-only sexual education on	31.2350	3.5843	2000	-0.122**	0.000	Sig.
Religion	01.0800	0.3500				

*No of participants.

**Sig. at 0.01 level.

***Pearson’s.

****'p' value.

**Null hypothesis 4:** There will be no joint effect of independent variables (age, gender, religion, ethnicity and parent’s occupation) on perspective of abstinence-only sexuality education.

### Was there any joint effect of independent variables (age, gender, religion, ethnicity and parent’s occupation) on perspective of abstinence-only sexual education?

A significant joint effect existed between the independent variables (age, gender, religion, ethnicity and parent’s occupation) and perspective on abstinence-only sexual education (F (5, 1994) = 13.085; R = 0.178, R ^2^ = 0.032, Adj. R ^2^ = 0.029; P < 0.05) ( Table 13). About 3% of the variation was jointly accounted for by the independent variables. The null hypothesis is therefore rejected.

**Table 13.** Joint effect of independent variables (age, gender, religion, ethnicity and parent’s occupation) on perspective of abstinence-only sexual education.

Model	Sum of squares	DF*	Mean square	F**	Sig.
Regression	815.864	5	163.173	13.085	0.000
Residual	24865.686	1994	12.470		
Total	25681.550	1999			

R = 0.178.

R ^2^ = 0.032.

Adj R ^2^ = 0.029.

*Degree of freedom.

**F-ratio.

### Would there be any relative effect of independent variables (age, gender, religion, ethnicity and parent’s occupation) on perspective of abstinence-only sexuality education?

There is a relative contribution of each of the independent variables on the dependent: age (β = 0.115, P < 0.05), gender (β = 0.042, P > 0.05), religion (β = -0.117, P < 0.05), ethnicity (β = -0.016, P > 0.05) and Parent’s occupation (β = 0.021, P > 0.05) ( Table 14). Hence, while age and religion made a significant contribution, gender, ethnicity and parent’s occupation were not significant.

**Table 14.** Relative contribution of independent variables (age, gender, religion, ethnicity and parent’s occupation) to perspective on abstinence-only sexual education.

Model	Unstandardised coefficient		Standardised coefficient	T	Sig.
	B	Std. error	β		
(Constant)	30.422	0.488		62.369	0.000
Age	0.705	0.136	0.115	5.168	0.000
Gender	0.319	0.166	0.042	1.918	0.055
Religion	-1.187	0.225	-0.117	-5.269	0.000
Ethnicity	-5.179E-02	0.073	-0.016	-0.714	0.475
Parent’s occupation	5.77E-02	0.061	0.021	0.954	0.340

### Would there be any significant relationship between perspective of abstinence-only sexual education and age, gender, religion, ethnicity and parent’s occupation?

A positive significant relationship existed between perspective on abstinence-only sexual education and age, a negative significant relationship between perspective of abstinence-only sexual education and religion but no relationship between perspective of abstinence-only sexual education and gender, ethnicity and parents’ occupation ( Table 15).

**Table 15.** Correlation matrix showing the relationship between perspective of abstinence-only sexual education and age, gender, religion, ethnicity and parent’s occupation.

	Abstinence-only sexual education	Age	Gender	Religion	Ethnicity	Parent’s occupation
Abstinence-only sexual education	1					
Age	0.123**	1				
Gender	0.051*	0.083**	1			
Religion	-0.122**	-0.020	0.007	1		
Ethnicity	-0.022	0.055*	0.034	0.128**	1	
Parent’s occupation	0.039	0.117**	0.021	-0.031	0.039	1
Mean	31.2573	2.22	1.65	1.08	2.21	2.20
S.D	3.5702	0.58	0.48	0.35	1.11	1.33

**Sig. at 0.01 level.

*Sig. at 0.05 level.

## Discussion

Young age is an important factor in the success of abstinence-only sexual education. According to Massey, three significant periods exist where values are learnt. They are the imprint period, modelling period and socialisation period ^48^.

During the imprint and modelling periods, children learn through instructions and modelling. During these periods, the behaviour of children is formed from instructions given to them and examples before them. Therefore, if abstinence-only sexual education is taught between the imprinting to modelling period, it might produce positive results in adolescent lives. During the period of socialisation which starts from 13–21 years, a child is already exposed to different types of views and influences. Introducing abstinence-only sexual education might not be successful at this stage because for several reasons. Probably, most of the adolescents would have become sexually active by this age. Secondly, they might have a conviction already for sexual intercourse outside the confines of marriage as an ideal way of life. It is possible to accept instructions at a younger age. Those within the age range of 11–13 years were 8.8% (N = 176) of the total population and they were the youngest group in this study. Most of them would have constituted the population in senior secondary one. The largest number of respondents was within the age bracket of 14–19 years. They might have established their beliefs about premarital sex and are probably sexually active already. This could be an explanation for the larger number of respondents that opposed abstinence-only sexual education.

The major finding of this study shows that Nigerian secondary school adolescents generally have a negative perspective towards abstinence-only sexual education. Out of a total population of 2000 respondents, abstinence-only sexual education was accepted by only 314 respondents and rejected by 1686. Those that had the tendency to accept abstinence-only sexual education were within the age bracket of 11–13 years and are the youngest group of adolescents The highest number of those that advocated for sex to be saved until marriage and also wanted abstinence-only sexual message and education belonged to the youngest group of adolescents. A greater number of respondents that were not in favour of abstinence-only sexual education belong to the 14–19 years age bracket and are the older adolescents. These are the ones within the age of socialisation already. With their exposure to different views about premarital sex, it is likely that most of them are already sexually active and have already taken a position for premarital sex. The message of abstinence-only sexual education at this stage might not be accepted with ease.

Religion had a negative significant influence on the adolescents' perspective on abstinence-only sexual education with the highest number of respondents being christians. Previous studies revealed that almost no religion supported premarital sexual activities. The studies of Concerned Women for America, demonstrated that religion acts as a deterrent to early sexual activity ^5^. Many adolescents in previous studies posited that morals, values and religious beliefs significantly influence the decision of whether to have sex ^3– 5^. Probably, those that accepted abstinence-only sexual education would have been influenced by their religious beliefs. This also implies that teaching of religious values could be a useful tool for inculcating the values of sexual intercourse within the confines of marriage. Fortunately 93.9% (N = 1877) of the total respondents were Christians 4.3% (N = 86) were Muslims and 1.6% (N = 31) belonged to traditional religion. Only 0.3% (N = 6) were pagans. Christianity should therefore teach abstinence-only sexual education period.

A greater number of male and female Nigerian secondary school adolescents generally have a negative perspective towards abstinence-only sexual education. Out of the number that favoured abstinence-only sexual education, more females than males were in favour of some statements period, in other statements more males than females were in agreement. Surprisingly, more males than females advocated for sex to be saved until marriage and also wanted abstinence-only sexual message and education. One would have expected a more positive response from the females than the males because a greater proportion of the respondents in this study were females 64.9% (N = 1298) of the total respondents. This finding is also sad because it is the females that suffer most from the adverse effects of premarital sexual activities. They are the ones that drop out from schools in the advent of pregnancy. They suffer the adverse effects of early pregnancy and child birth or abortion so one would have expected more females than males to advocate for sex to be saved until marriage. At the other hand more males than females advocating for sex to be saved until marriage and also wanting strong abstinence message and education might not be unconnected with the high number of males among the 11–13 years adolescents ( Table 16). This study has revealed age 11–13 years as the group with a more likelihood of accepting abstinence-only sexual education than the other two groups of adolescents.

**Table 16.** Descriptive analysis of age and gender distribution.

Age	Gender				Total	
	Male		Female			
	N	(%)	N	(%)	N	(%)
11–13 years	105	59.7%	71	40.3%	176	100.0%
14–16 years	389	31.8%	834	68.2%	1223	100.0%
17–19 years	208	34.6%	393	65.4%	601	100.0%
Total	702	35.1%	1298	64.9%	2000	100.0%

Ethnicity did not make any significant contribution to perspective of adolescents on abstinence-only sexual education. This shows that adolescents are all the same anywhere and everywhere. This study also revealed that about 80% of the total respondents could not define sexual education. Probably most of the responses would have been informed by ignorance.

## Conclusion

This study therefore concludes that young adolescents within the age bracket of 11–13 years demonstrated the likelihood of being more receptive to abstinence-only sexual education. This finding suggests the need to teach abstinence-only sexual education at an early age by parents who are the first contacts, in religious organizations by sunday school teachers and then by teachers in schools where abstinence-only sexual education is preferred. This will in turn form a part of the values and belief system of the child which might not be easily compromised.

## Translations to health education practice

Knowing the perspective of adolescents on abstinence-only sexual education is the key to knowing the right intervention programme to design and the approach to adopt for the implementation of such programme. This study reveals that abstinence-only sexual education will not work for majority of the respondents because it is not acceptable to them. For instance, only 314 out of 2000 respondents agreed with statements reflecting acceptance of abstinence-only sexual education while 1686 respondents disagreed. A very important finding of this study is that abstinence-only sexual education might only impact positively on those within the imprint and modelling periods of development. This includes those within the age range of 11–13 years. Those within the age bracket of 14–19 years seem to have formed their opinion already from different socialisation processes. This finding suggests the need for a second study only for those within the imprint and modelling period. A new study should address the efficacy of abstinence-only sexual education and the willingness by the young adolescents to practice messages of abstinence-only sexual education. The intervention programme based on the findings of this study recommends a two-dimensional approach. Comprehensive sexual education for the older age group (14–19 years) and abstinence only for the younger ones (11–13 years).

The implications of this study are directed specifically to parents who are the primary caretakers of the children. They have the first contact with the children during the early years. The religious organisations and the school health educators are also very important role players. Findings from this study suggest the introduction of different programming strategies aimed at teaching abstinence-only sexual education as a way of life within the early ages. This might help in safeguarding adolescents especially the females from the numerous life threatening adverse effects associated with premarital sexual activities.

Parents must have a sound knowledge of sexual education so that they can serve as effective teachers to the children. This is the reason it is very good also to empower today’s adolescents with the knowledge of sexual education and the benefits as future parents. Preparing the adolescents of today to become knowledgeable parents of tomorrow might assist in creating a subsequent future of reduced cases of teenage pregnancies, deliveries, abortions and sexually transmitted infections. Whatever is wrong today can be corrected through adequately prepared adolescents who are the future of any nation.

Religious organisations should never relent in teaching the morals and values of abstinence-only sexual education to the youngest group of adolescents with the likelihood to accept. This should be commenced early enough right from their Sunday school classes with negative beliefs and design some remedial programmes for them to reduce the negative influence on other adolescents through the process of socialisation. It can be seen from this study that many problems faced by adolescents as a result of pre-marital sexual activity are avoidable.
