# Supplementary material for: Nigerian secondary school adolescents’ perspective on abstinence-only sexual education as an effective tool for promotion of sexual health
Source: F1000Res. 2013 Dec 4;2:86. Originally published 2013 Mar 13. [Version 2] doi: 10.12688/f1000research.2-86.v2 (PMC3924948; doi:10.12688/f1000research.2-86.v2)
Supplement: Peer review history

# Peer review: Referee response for version 2

**DOI:** 10.5256/f1000research.3146.r4008

**Published:** 2014-03-10

**Authors:** Laxmi Baxi

**Affiliations:** 1 Department of Obstetrics and Gynecology, NYU Langone Medical Center , New York, NY, USA

I have read this submission. I believe that I have an appropriate level of expertise to confirm that it is of an acceptable scientific standard.

# Peer review: Referee response for version 2

**DOI:** 10.5256/f1000research.3146.r2650

**Published:** 2013-12-11

**Authors:** Luis Bahamondes

**Affiliations:** 1 Department of Obstetrics and Gynaecology, School of Medical Sciences, Universidade Estadual de Campinas, Campinas, Brazil

I am comfortable with the modifications made by the authors and I have no further comments.

I have read this submission. I believe that I have an appropriate level of expertise to confirm that it is of an acceptable scientific standard.

# Peer review: Referee response for version 1

**DOI:** 10.5256/f1000research.1210.r970

**Published:** 2013-05-30

**Authors:** Luis Bahamondes

**Affiliations:** 1 Department of Obstetrics and Gynaecology, School of Medical Sciences, Universidade Estadual de Campinas, Campinas, Brazil

The authors submitted a manuscript in which they assessed the perspectives of Nigerian secondary school students on abstinence-only sexual education. They applied a questionnaire to obtain the information. The sample size is correct and the analysis of the data is well conducted. However, my specific comments are that the title does not reflect what the authors did, as the authors did not evaluate the efficacy of abstinence-only sexual education; in fact, there is no evidence of whether the abstinence-only sexual education changes the attitudes of the students. Furthermore, the comments on religion are inappropriate because most of the interviewed students practiced only one religion.

In my opinion, the following statement is strongly speculative and inappropriate because the authors did not obtain any evidence for it: “Preparing the adolescents of today to become knowledgeable parents of tomorrow can assist in creating a subsequent future of reduced cases of teenage pregnancies, deliveries, abortions and sexually transmitted infections. Whatever is wrong today can be corrected through adequately prepared adolescents who are the future of any nation.”

I have read this submission. I believe that I have an appropriate level of expertise to confirm that it is of an acceptable scientific standard, however I have significant reservations, as outlined above.

# Response

**Published:** 2013-06-04

**Authors:** Mfrekemfon Inyang

**Affiliations:** University of Port-Harcourt, Nigeria, Nigeria

Much thanks to the referee for the comments. In this study the authors only looked at the Nigerian secondary school adolescents' perspective on abstinence-only sexual education. The purpose of this study is also clearly stated. The authors have not indicated anywhere in the study of evaluating the efficacy of abstinence-only sexual education, but only intend to do that in a follow up study. This study is not an intervention study so it could not have shown if abstinence-only sexual education could change attitude. It is always necessary to clarify needs before following up with appropriate intervention. The intent for a follow up intervention study has been indicated. The aspect of religion has been earlier addressed. The statement is a recommendation to educate the adolescents with the knowledge of what is right or wrong early in life. Numerous studies have proven the transforming power of information. It has also been established that most of the wrong-doings of adolescence are either due to wrong information or outright ignorance.

Thanks.

Dr. M. P. Inyang

# Peer review: Referee response for version 1

**DOI:** 10.5256/f1000research.1210.r971

**Published:** 2013-05-30

**Authors:** Laxmi Baxi

**Affiliations:** 1 Department of Obstetrics and Gynecology, NYU Langone Medical Center , New York, NY, USA

- The title gives the impression that it refers to the entire population; however, it is true only for the youngest group - further clarification is needed.
- The majority of individuals in this study are Christian, so comparison by religion does not appear to be adequate. The authors need to emphasise this and may describe such findings in a single statement.
- The manuscript is too long, as is the introduction. It should be shortened to ensure the reader does not lose interest.
- Several text descriptions should be omitted as tables are self explanatory, e.g. table 1.
- Furthermore, it should be mentioned that the data only refers to a particular group of the population.

I have read this submission. I believe that I have an appropriate level of expertise to confirm that it is of an acceptable scientific standard, however I have significant reservations, as outlined above.

# Response

**Published:** 2013-06-04

**Authors:** Mfrekemfon Inyang

**Affiliations:** University of Port-Harcourt, Nigeria, Nigeria

Much thanks to the referee! The title of the work is what was studied. Title of the work is different from findings.These findings are clearly spelt out in the work.The religion aspect has been addressed already. Other observations are all addressed accordingly.

Thanks.

Dr. M. P. Inyang

# Peer review: Referee response for version 1

**DOI:** 10.5256/f1000research.1210.r877

**Published:** 2013-04-08

**Authors:** Warren Foster

**Affiliations:** 1 McMaster University, Hamilton, Canada

The authors examined secondary school student perspectives on abstinence-only sexual education through the use of a questionnaire. The sample size is robust, the statistical methods are appropriate, and overall the paper is well written. Unfortunately, the title of the paper is misleading. Specifically, the authors only examined student perspectives and there were no measures of efficacy of abstinence-only sexual education. The data suggests only that students of the youngest age group are more likely to be receptive to abstinence-only sexual education but no evidence is presented on whether the students would or in fact do incorporate these messages into their lives. The authors also suggest that the data shows that there is a significant relationship between religion and student perspective; however, the numbers of students in other religions than Christianity is too small to make meaningful conclusions. Moreover, it is unclear if the students simply identify with a particular religion or actively participate in the religion.  Finally, the conclusions reached and the implications for health education practice are overstated. The most that can be concluded from this study is that young adolescents are potentially more receptive to abstinence-only sexual education. It cannot be concluded that abstinence-only sexual education will be more or less effective than no education or alternative education messages in shaping student sexual practices.

I have read this submission. I believe that I have an appropriate level of expertise to confirm that it is of an acceptable scientific standard, however I have significant reservations, as outlined above.
